# Safety and immunogenicity of a live attenuated influenza H5 candidate vaccine strain A/17/turkey/Turkey/05/133 H5N2 and its priming effects for potential pre-pandemic use: a randomised, double-blind, placebo-controlled trial

**DOI:** 10.1016/S1473-3099(17)30240-2

**Published:** 2017-08

**Authors:** Punnee Pitisuttithum, Kobporn Boonnak, Supat Chamnanchanunt, Pilaipan Puthavathana, Viravarn Luvira, Hatairat Lerdsamran, Jaranit Kaewkungwal, Saranath Lawpoolsri, Vipa Thanachartwet, Udomsak Silachamroon, Wanibtisam Masamae, Alexandra Schuetz, Ponthip Wirachwong, Sit Thirapakpoomanunt, Larisa Rudenko, Erin Sparrow, Martin Friede, Marie-Paule Kieny

**Affiliations:** a Vaccine Trial Centre, Mahidol University, Bangkok, Thailand; b Center of Excellence for Biomedical and Public Health Informatics, Mahidol University, Bangkok, Thailand; c Faculty of Tropical Medicine, Mahidol University, Bangkok, Thailand; d Department of Microbiology, Faculty of Medicine, Siriraj Hospital, Mahidol University, Bangkok, Thailand; e Faculty of Medical Technology, Mahidol University, Bangkok, Thailand; f Department of Retrovirology, Armed Forces Research Institute of Medical Science, United States Component, Bangkok, Thailand; g Henry M Jackson Foundation for Advancement of Military Medicine, Bethesda, MD, USA; h The Government Pharmaceutical Organization, Bangkok, Thailand; i The Institute of Experimental Medicine, St Petersburg, Russia; j World Health Organization, Geneva, Switzerland

## Abstract

**Background:**

The emergence of highly pathogenic avian influenza H5N1 viruses has raised concerns about their pandemic potential. Vaccination is the most effective way of preventing influenza. In this study, we investigated the safety and immunogenicity of an avian H5N2 live attenuated influenza vaccine (LAIV H5N2) in healthy Thai adults and its priming immune responses with an H5N1 inactivated vaccine boost.

**Methods:**

This study was done at the Vaccine Trial Centre at Mahidol University, Bangkok, Thailand and was divided into two parts. Part 1 consisted of a randomised, double-blind, placebo-controlled trial done over 18 months. We randomly assigned (2:1) healthy Thai adults aged 18–49 years with a computer generated randomisation sequence (blocks of six) to receive either two intranasal doses (0·25 mL per nostril) of LAIV H5N2 (101 participants) or placebo (51 participants) 21 days apart. For part 2, an open-label trial was done in which previously vaccinated participants (40 from LAIV H5N2 group and 20 placebo) were given one intramuscular dose (0·5 mL) of H5N1 booster vaccine. Participants, investigators, and site-study workers were blinded from randomisation. Immune responses after subsequent immunisation were evaluated using haemagglutination-inhibition and microneutralisation assays and circulating follicular T-helper cells and plasmablast cells were measured in serum and whole blood. The trials are registered with ClinicalTrials.gov, numbers NCT01841918 and NCT02229357.

**Findings:**

Between Feb 4, 2013, and Feb 28, 2013, 256 individuals were screened, of whom 152 participants were enrolled in part 1 of this study. LAIV H5N2 vaccine was well tolerated. Viral shedding was detected in only six (6%) of 101 participants in the vaccine group 1 day after the first vaccination and in and two (2%) of 98 participants in the group after the second vaccination. There was no serious adverse event in both groups. 51 (50%) of 101 participants in the vaccine group and 28 (55%) of 51 in the placebo group reported at least one adverse event. 80 (84%) of 95 events in the vaccine group and 32 (78%) of 43 events in the placebo groups were reportedly suspected adverse events, probably related to the vaccine; however, most were mild in nature. After two doses of vaccine, 13 (13%) of 100 participants in the vaccine group had an increase in haemagglutination-inhibition titre of more than four-fold and four (4%) of 100 vaccinees developed a rise in neutralisng antibody titre of more than four-fold. 1 year later, after a booster with an inactivated H5N1 vaccine (part 2), 39 (98%) of 40 participants who had previously been vaccinated with LAIV H5N2 had an increase in haemagglutination-inhibition titre of greater than four-fold as early as day 7 compared with three (15%) of 20 participants in the placebo group. Peak geometric mean titre (GMT) for haemagglutination-inhibition antibodies in the previously LAIV H5N2 vaccinated group (566·89 [95% CI 436·97–735·44]) were significantly higher than among those who previously received placebo (25·49 [11·82–54·96]; p<0·0001). The peak GMT by neutralising antibody assay in the H5N2 vaccinated group (1395·85 [1040·79–1872·03]) was also significantly higher than that observed in the placebo group (17·41 [9·05–33·48]; p<0·0001). Importantly, higher cross-reactive haemagglutination-inhibition antibody titres against H5N1 (clades 1, 2.1.3.2, and 2.3.4) were detected in the LAIV H5N2 experienced group than the naive group (p<0·0001).

**Interpretation:**

Our data suggest that LAIV vaccination induces long-lasting memory immune responses. The limitation of this study was that part 2 was designed as a proof-of-concept study by contrast with part 1.

**Funding:**

WHO.

**Research in context**

**Evidence before this study**

We searched PubMed for articles published in English before Sept 14, 2016, with the search terms “H5 vaccine candidates and LAIV” and “clinical trials and H5 vaccine”. Our search found a very limited number of studies on LAIV H5 viruses. Because this study was focused on the safety and immunogenicity of LAIV H5N2 influenza vaccine, we included only those studies similar to our work using live attenuated H5 vaccine candidates. Immunogenicity of an LAIV H5N2 vaccine candidate has been determined in several studies; however, only two studies investigated immunological priming effects of H5 LAIV vaccine candidates. The vaccine candidates were safe, well tolerated, and immunogenic in the adult population, and long-lasting immune memory responses with cross-reactivity were also observed.

**Added value of this study**

To our knowledge, no such priming and boosting strategy has been done in countries where the H5 virus is potentially circulating, such as Thailand. We showed a strong immune response to both H5N2 and H5N1 avian influenza viruses when participants were vaccinated with two doses of LAIV H5N2 vaccine candidate followed by boosting with H5N1 inactivated vaccine. Although two doses of LAIV H5N2 vaccine were poorly immunogenic as measured by haemagglutination-inhibition assay, the priming effect of LAIV H5N2 was unmasked by boosting with H5N1 inactivated vaccine. High titre of antibodies and broadly reactive and rapid antibody responses after boosting vaccination with inactivated H5N1 vaccine were detected in most previously received LAIV H5N2 participants. LAIV induced long-lasting memory responses.

**Implications of the available evidence**

Our vaccination strategy could be used to prime a population during the early pandemic period, after which a booster dose could be given during an epidemic, if needed. However, further investigation into the effectiveness of this strategy is needed when it is used in the field.

## Introduction

Avian influenza viruses are spreading rapidly in bird populations. Some highly pathogenic avian influenza (HPAI) viruses can cause severe respiratory disease and death in human beings. Since the first recorded direct bird-to-human transmission of HPAI H5N1 in Hong Kong in 1997,^1^ the HPAI H5 viruses have spread to Africa, Asia, Europe,^2^ the Middle East, and North America.^3^ From 2003 to 2015, WHO recorded 844 confirmed human cases of H5N1 infection in 16 countries and 449 deaths.^4^ In Thailand, a 68% case-fatality rate for H5N1 virus infection was reported in 2004–06,^5^ as well as two cases of probable person-to-person transmission of the virus.^6^

The spread of H5N1 viruses has raised concerns about their potential to cause pandemics. In response to this potential threat, several H5N1 vaccine candidates have been prepared and evaluated in animal models and clinical trials. Live attenuated influenza vaccines (LAIVs) generally induce moderate antibody responses. However, the poor immunogenicity of the haemagglutinin proteins of HPAI H5N1 viruses has posed challenges for H5 LAIV development. For example, a subvirion vaccine derived from low pathogenicity influenza A/duck/Singapore/97 virus H5N3 (which is antigenically related to the HPAI H5N1 virus that emerged in Hong Kong in 1997) was not immunogenic in human beings in a phase 1 trial.^7^ In 2006, a monovalent A/Vietnam/1203/2004 H5N1 subunit vaccine was well tolerated, but only modestly immunogenic, in a phase 1 clinical trial.^8^ A candidate H5N1 A/Vietnam/1203/2004 LAIV, based on the A/Ann Abor/6/60 master donor virus, was developed in the USA.^2^ The vaccine highly restricted replication in human beings and induced only low-titre haemagglutination-inhibition and neutralising serum antibody responses, even after two doses given by nasal spray.^9^ An H5N2 LAIV was developed in Russia using the A/Leningrad/134/17/57 master donor virus, and the haemagglutinin was derived from A/duck/Potsdam/1402–6-86 H5N2.^1^ The same vaccine was evaluated in small phase 1–2 clinical trials^10^ in Russia: two doses of the vaccine induced seroresponses (four-fold or greater rise in titre) to the homologous virus in 47–55% of participants. Evidence of cross-reactive antibodies against A/Indonesia/05/2005 H5N1 was also observed in 29–31% of participants. Phase 1 trials of an A/turkey/Turkey/05/133 H5N2 vaccine candidate in Russia showed that the vaccine was safe and well tolerated, and elicited modest immunogenicity in healthy adults.^11^

The immunogenicity of H5 traditional inactivated vaccines and LAIVs is not robust as measured by haemagglutination-inhibition and microneutralisation assays. Several alternative approaches have therefore been tested, including addition of adjuvants, increased vaccine doses, and different combinations of vaccines in prime–boost regimens.^12^ Promising results have been obtained from clinical trials of various prime–boost regimens of H5N1 vaccines: recombinant haemagglutinin vaccine followed by inactivated vaccine,^13^ DNA vaccine followed by inactivated vaccine,^14^ vectored vaccine expressing H5 haemagglutinin followed by inactivated vaccine,^15^ and LAIV followed by inactivated vaccine.^16^

We did a study composed of two parts. In part 1, we evaluated immune responses and safety of LAIV H5N2 candidate strain A/17/turkey/Turkey/05/133. In part 2, we compared the immunogenicity of the inactivated H5 influenza vaccine single dose among study participants who in part 1 were vaccinated with LAIV H5N2 versus naive participants (previously received placebo). The main hypothesis for part 2 was that the administration of LAIV H5N2 would prime the immune system for an efficient memory response to an inactivated H5N1 influenza vaccine.

## Methods

### Study design and participants

Part 1 was a randomised, double-blind, placebo-controlled study to assess the safety and reactogenicity of an H5N2 LAIV. We assessed the seroconversion rate postvaccination based on serum hemagglutination-inhibition antibodies and neutralising antibodies. This study was done at the Vaccine Trial Centre, Faculty of Tropical Medicine, Mahidol University, Bangkok, Thailand, which is a 30-bed isolation ward with self-contained air conditioning and drainage system. For part 2, 60 participants were give one dose of inactivated H5N1 vaccine in a non-randomised open-label study to compare the immune responses between those previously vaccinated with LAIV H5N2 and placebo groups. Participants were healthy Thai men and women aged 18–49 years with an antibody titre to specific H5 influenza virus of less than 1/40 by haemagglutination inhibition. Characteristics of participants were similar to those reported previously.^11^, ^17^ Breastfeeding mothers, pregnant women, and women who planned to become pregnant within 60 days of enrolment were excluded from the study.

This study was approved by the ethics review committees of the Faculty of Tropical Medicine, Mahidol University and of WHO. Written informed consent was obtained from all participants before any study-related activities.

### Randomisation and masking

For part 1, study participants were randomly assigned (2:1) to either the vaccination group or the placebo group. The randomisation list was generated by an independent statistician as a block of six. The randomisation was kept in a secured safe box at the centre for data management and analysis. Only designated people were authorised to access the randomisation codes. However, complete randomisation blocks of treatment materials were sent only to the manufacturer for study vaccine preparation and distribution to study sites. All other study investigators as well as the volunteers were be blinded to the randomised codes. The emergency unblinding process could be done only when there was a special request for ethical and emergency clinical concerns by the sponsor or ethical committee or the data safety monitoring board. For part 2, all participants who were contactable throughout the study were given boosting vaccine.

### Procedures

For part 1, participants were given two doses of candidate LAIV strain A/17/turkey/Turkey/05/133 H5N2 or placebo by the intranasal route, the first dose on day 1 and the second on day 28. All participants were kept in an isolation ward for 5 days after each immunisation to assess safety and were discharged only after a negative nasal swab culture. Participants who had a positive nasal swab on day 3 by PCR were given oseltamivir (150 mg twice per day for 5 days). All participants were closely monitored for any adverse reactions by a qualified physician and nurses.

The follow-up period was 60 days. We collected blood on days 1 and 28, before each immunisation, and on days 49 and 60, to measure immune responses by haemagglutination inhibition, microneutralisation, and serum IgG and IgA ELISA. We did routine laboratory safety investigations on days 28 and 49. We took nasal swabs on days 2, 3, and 5 to assess viral shedding by RT-PCR and virus isolation in chicken embryonated eggs using the WHO protocol.^18^

In part 2 of the study, after 1 year, 60 participants were contacted serially according to the study number previously allocated (40 participants from the LAIV H5N2 vaccinated group and 20 from the placebo group) and were given a single 0·5 mL intramuscular dose of an inactivated H5N1 influenza vaccine on day 1. Participants were given a diary card to record any reactogenicity and adverse events at home for 3 days. We collected blood specimens before immunisation on day 1, and on days 7, 28, and 90 to test for haemagglutination-inhibition antibody titre, neutralising antibody titre, circulating follicular T-helper cells (T_FH_), and plasmablasts.

The study vaccine for part 1 was a live attenuated influenza H5N2 vaccine candidate strain, A/17/turkey/Turkey/05/133. The vaccine strain was produced using classic genetic reassortment in chicken embryos. The donor strain was A/Leningrad/134/17/57 H2N2, which has cold-adapted and temperature-sensitive properties. The candidate strain contained haemagglutinin from A/turkey/Turkey/1/05 H5N1. The cold-adapted, temperature-sensitive, attenuated vaccine candidate strain was prepared at the Institute of Experimental Medicine (St Petersburg, Russia)^11^ and was manufactured by the Government Pharmaceutical Organization (Thailand). The liquid formulation of H5N2, at 7·5–8·5 log 50% embryo infectious dose (EID_50_) per 0·5 mL dose, was administered intranasally at 0·25 mL per nostril, using a sterile syringe and nozzle sprayer. The formulation contained 6·8% sucrose, 1% porcine hydrolysed gelatin, 1·2% arginine, 0·1% glutamate, 1·1% dipotassium phosphate, and 0·5% monopotassium phosphate. The vaccine was stored at −20°C (±5°C). The placebo consisted of the above composition without the vaccine virus with the same volume and procedure. Two doses of vaccine were given 28 days apart.

The booster vaccine was OrniFlu, an inactivated subunit adsorbed influenza vaccine (Federal State Scientific-Industrial Company Microgen for Immunobiological Medicines, Ministry of Health, Russia) obtained from purified H5N1 vaccine seed strain containing *HA* and *NA* genes of the influenza virus A/turkey/Turkey/1/2005 H5N1 (NIBGR-23) and *PA, PB1, PB2, NP, M*, and *NS* genes from the A/PR/8/34 strain. The vaccine contains haemagglutinin and neuraminidase isolated from purified virions of type A avian influenza virus with serotype H5N1, grown in chicken embryos and adsorbed onto aluminium hydroxide and thiomersal as stabilising agent. The vaccine was stored at 2–8°C.

We extracted viral RNA from nasal swabs. We did RT-PCR assays with primers targeting the *M* gene of the virus to detect LAIV H5N2 virus. We also tested the swabs by inoculation in 10–11-day-old embryonated chicken eggs. We sequenced the virus obtained from inoculated embryonated chicken eggs to detect viral mutation by sequence analysis.

We treated serum samples with receptor-destroying enzyme and did two-fold serial dilutions in 96-well plates, starting at a dilution of one in ten. We added phosphate-buffered saline alone or virus in the absence of antibody to control wells. The virus and serum were incubated together at room temperature for 30 min, and 50 μL of 0·5% (v/v) horse erythrocytes added. The antibody, virus, and erythrocytes were gently mixed, and the results recorded after incubation for 45–60 min at room temperature. Haemagglutination-inhibition titres were recorded as the inverse of the highest antibody dilution that inhibited haemagglutination. The viruses used in this assay were A/17/turkey/Turkey/05/133 H5N2, clade 2.2.1, rg-H5N1-KAN-1, which contained haemagglutinin and neuraminidase of A/Thailand/1(KAN-1)/04 H5N1, clade 1 and *PA, PB1, PB2, NP, M*, and *NS* genes from A/PR8/34 H1N1, A/Indonesia/5/2005 H5N1 (PRxCDC-RG), clade 2.1.3.2, and reverse genetic virus containing haemagglutinin from A/Laos/Nong Khai/1/2007 H5N1, clade 2.3.4.

The neutralising antibody titre was expressed as a value reciprocal to the highest dilution of a sample that yields a 50% reduction in the amount of viral nucleoprotein produced in the infected cultures compared with the virus control. The viruses tested were the same as those tested in the haemagglutination-inhibition assay. Seroconversion was measured as a four-fold increase in antibody titres in any of the above assays before and after vaccination.

For analysis of inducible costimulator (ICOS)+ T-cell, whole blood samples (500 μL) were processed and stained with antibodies against cell-surface markers CXCR5, CD3, CD8, CD4, CCR6, CXCR3, ICOS, CD45RA, and CD45 (BD Bioscience, San Jose, CA). For plasmablast analysis, whole blood samples (500 μL) were processed and stained with antibodies against the specific cell-surface markers CD27, CD19, CD20, and CD38. Cells were analysed using flow cytometer BD LSRII with a four-laser configuration (BD Bioscience, San Jose, CA) and data were analysed using Flow Jo (version 9.7.5; TreeStar, Ashland, OR).

We measured IgG and IgA antibodies in serum samples and in nasal wash samples by ELISA, as described previously,^17^ using recombinant H5N1 proteins (obtained from National Institute for Biological Standards and Control, UK). Positive IgA was established based on the cutoff value (optical density ≥0.2) established from the average optical density values obtained from the prevaccination samples plus 3 SD.

Clinical evaluations included grading of reporting temperature, and local and systemic reactions were assessed daily for 5 days after immunisation and all adverse events were collected after first immunisation throughout the period of the study.

### Outcomes

For part 1, the primary outcomes were seroconversion against vaccine strain of influenza virus analysed by haemagglutination-inhibition and microneutralisation assays, safety in terms of the reactogenicities or adverse events reported, and viral shedding and virus genetic stability. The secondary outcome was concentration of serum IgG, serum IgA, and nasal IgA.

For part 2, the primary outcomes were seroconversion against H5 strains analysed by haemagglutination-inhibition and microneutralisation assays. The secondary outcomes were injection site and systemic reactogenicity adverse events to the H5 inactivated influenza vaccine, and immunogenicity (defined as increasing plasmablast B-cell count and ICOS+ T-cell count in participants previously primed with LAIV H5N2).

### Statistical analysis

For part 1 of the study, the sample size of 150 (100 vaccine *vs* 50 placebo) was based on the confidence interval of the comparison of the two groups. Assuming that there was no seroconversion in the placebo group and 70% seroconversion in vaccine group, with expected two-sided 95% CIs of 60·8–79·2%, a sample size of 50 in the placebo and 100 in the vaccine group was needed. This sample size calculation was based on the European guidelines for influenza vaccine trials.^19^ For adults aged 18–49 years, to detect at least 0·1% of adverse reactions, a minimum of 50 immunologically naive individuals were needed for each dose or regimen to identify acceptable formulations and schedules.^18^ The statistical power of part 2 of the study was retrospectively calculated because it was an exploratory proof-of-concept analysis. Based on the percentage of participants with four-fold increase in haemagglutination-inhibition antibody of 97·5% in 40 vaccinated participants and 15·0% in 20 naive, the power (1 –** **β) to detect the difference of immune response at day 7 after boosting between the two groups was 1·0.

We analysed the data by the intention-to-treat (ITT) principle, defined as all individuals who are randomly assigned to either study group. The ITT analysis included individuals who did not comply with the protocol-defined treatment schedule—ie, individuals who did not have the vaccination and complete follow-up.

For safety analysis, all participants who received at least one dose of vaccine were included, whereas for humoral analysis, those participants who had at least one evaluable endpoint were included. The primary endpoints of immune responses were calculated based on a four-fold or greater increase in haemagglutination-inhibition and microneutralisation antibody titre after vaccination. Geometric mean titres (GMTs) were assessed by the differences in titres observed on day 1 (prevaccination), day 7, day 28, and day 90 after vaccination. The secondary endpoints were analysed as increasing numbers of T_FH_ cells and plasmablast B cells after vaccination. We analysed differences in immune responses and safety variables between the two groups with the significance level of p<0·05. We used the χ^2^ statistic or Fisher's exact test to assess differences in two binomial proportions, Student's *t* test or Wilcoxon rank sum test to assess differences in continuous data, and Spearman's correlation to establish the relationship between T_FH_ cell count and antibody titre.

These trials are registered with ClinicalTrials.gov, numbers NCT01841918 and NCT02229357.

### Role of the funding source

The sponsor of the study had no role in study design, data collection, data analysis, data interpretation, or writing of the report. The corresponding author had full access to all the data in the study and ES, MF, and M-PK had final responsibility for the decision to submit for publication.

## Results

**Figure 1:**
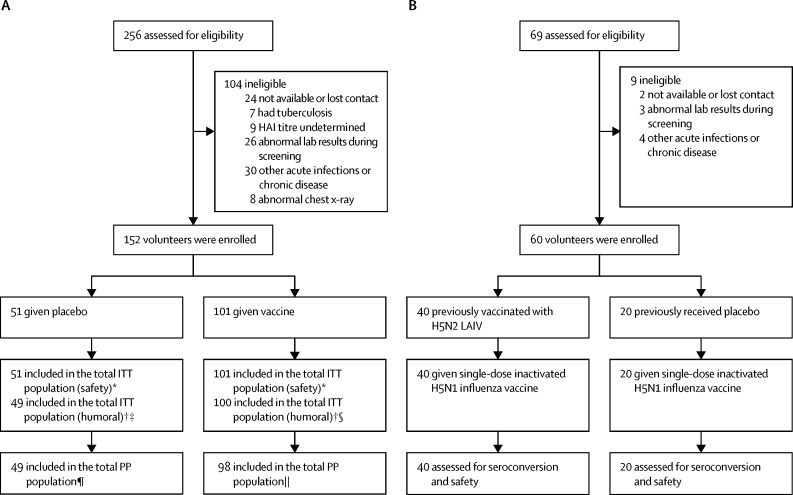
Trial profile Overview of participants who received LAIV H5N2 vaccine or placebo (A) and those who subsequently received H5N1 inactivated influenza vaccine (B). HAI=haemagglutination inhibition. ITT=intention-to-treat. LAIV H5N2=H5N2 live attenuated influenza vaccine. *Participants who had received at least one dose of LAIV H5N2 vaccine (n=51) were included in ITT analysis for safety. †Participants who had received two doses of LAIV H5N2 vaccine (n=49) were included in ITT analysis for immune response. ‡Humoral responses in two participants were obtained only on day 1 (before vaccination): one withdrawn by physician; one did not provide blood sample for analysis. §Humoral response in one participant was obtained only on day 1 (before vaccination); participant voluntarily withdrew. ¶One participant withdrawn by physician, one lost to follow-up. ||Two participants did not receive second immunisation; one voluntarily withdrew.

Between Feb 4, 2013, and Feb 28, 2013, 256 individuals were screened, of whom 152 participants were enrolled in part 1. 104 participants were excluded after the screening because of abnormal laboratory tests, abnormal chest x-rays, and medical history. 101 of 152 enrolled participants were randomly assigned to receive two doses of LAIV H5N2 and 51 participants were assigned to receive placebo (figure 1A). There were no substantial differences between the two groups in terms of demographic profile (table 1).

**Table 1:** Demographic characteristics of participants in the modified intention-to-treat population

		**Part 1**		**Part 2**	
		Vaccine (n=101)	Placebo (n=51)	Vaccine (n=40)	Placebo (n=20)
Sex					
	Male	39 (39%)	21 (41%)	14 (35%)	7 (35%)
	Female	62 (61%)	30 (59%)	26 (65%)	13 (65%)
Age (years)		30·87 (8·68)	32·07 (8·30)	35·18 (9%)	33·80 (8%)
Height (cm)		162·1 (8·29)	162·2 (7·23)	160·7 (8%)	159·8 (6%)
Weight (kg)		59·10 (9·13)	58·74 (9·90)	60·51 (11%)	58·92 (7%)
Birthplace					
	Bangkok and suburbs	65 (64%)	27 (53%)	26 (65%)	11 (55%)
	Other	36 (36%)	24 (47%)	14 (35%)	9 (45%)
Occupation					
	No occupation	6 (6%)	3 (6%)	3 (8%)	3 (15%)
	Student	20 (20%)	10 (20%)	5 (12%)	5 (25%)
	Government officer	0	0	0	0
	Employed	55 (54%)	23 (45%)	23 (58%)	6 (30%)
	Other	20 (20%)	15 (29%)	9 (22%)	6 (30%)
Education					
	No education	0	0	0	0
	Primary school	12 (12%)	4 (8%)	4 (10%)	3 (15%)
	Secondary school	46 (46%)	23 (45%)	12 (30%)	11 (55%)
	Vocational	11 (11%)	6 (12%)	6 (15%)	2 (10%)
	Bachelor degree	30 (30%)	18 (35%)	17 (43%)	4 (20%)
	Higher than bachelor degree	2 (2%)	0	1 (2%)	0

Data are n (%) or mean (SD).

1 year after vaccination, 60 of 69 participants who had received the LAIV H5N2 vaccine in part 1 were enrolled for part 2 (figure 1B). Nine participants were excluded after screening because of health reasons (such as abnormal lab findings during screening and other acute infections or chronic dieases); 40 participants had received two doses of LAIV H5N2, and 20 had received placebo. There were no substantial differences in baseline demographics between the two groups (table 1).

The LAIV H5N2 vaccine appeared to be safe; no serious adverse events were recorded in either group. 51 (50%) of 101 participants in the vaccine group and 28 (55%) of 51 participants in the placebo group reported at least one adverse event. 80 (84%) of 95 events in the vaccine group and 32 (78%) of 43 events in the placebo group were reported as probably related to vaccination. Most adverse events were mild. The most frequent local reactions following the first vaccination were runny nose (ten [10%] of 101 participants in the vaccine group), redness of the nose (nine [9%]), and nasal congestion (nine [9%]; table 2). Post-nasal drip was the most common systemic reaction in both treatment groups (table 2). Most reported local and systemic symptoms were less frequent after the second vaccination than after the first vaccination (table 2).

**Table 2:** Local and systematic reactogenicities reported in participants received LAIV H5N2 or placebo in the modified ITT population in part 1

	**First vaccination**			**Second vaccination**		
	Vaccine (n=101)	Placebo (n=51)	p value	Vaccine (n=98)	Placebo (n=49)	p value
Local reaction*						
Congested nose	9 (9%)	2 (4%)	0·336	6 (6%)	0	0·179
Runny nose	10 (10%)	2 (4%)	0·339	8 (8%)	3 (6%)	0·752
Shortness of breath	0	0	..	1 (1%)	0	1·00
Sore throat	6 (6%)	3 (6%)	1·00	5 (5%)	3 (6%)	1·00
Bad taste in mouth	2 (2%)	3 (6%)	0·335	0	0	..
Burning sensation in nose	3 (3%)	2 (4%)	1·00	0	0	..
Redness of nose	9 (9%)	4 (8%)	1·00	0	0	..
**Systemic reaction**†						
Headache	9 (9%)	2 (4%)	0·336	6 (6%)	2 (4%)	0·719
Chills	0	0	..	0	0	..
Myalgia	3 (3%)	0	0·551	0	0	..
Arthralgia	0	0	..	0	0	..
Fatigue	0	0	..	0	0	..
Post-nasal drip	17 (17%)	10 (20%)	0·672	4 (4%)	2 (4%)	1·00
Poor appetite	0	0	..	0	0	..
Diarrhoea	1 (1%)	0	1·00	1 (1%)	1 (2%)	1·00
Rash	2 (2%)	0	0·551	0	0	..
Urticaria	0	0	..	0	0	..
Cough	5 (5%)	3 (6%)	1	5 (5%)	2 (4%)	1·00
Tiredness on exertion	0	0	..	0	0	..
Nausea	3 (3%)	0	0·551	0	0	..
Vomiting	0	0	..	0	0	..

Data are number of participants (%). ITT=intention-to-treat.

* Any local reaction on any day.

† Summary by case who had symptoms.

H5N2 virus in nasal swab samples was detected by PCR assay in 56 (55%) of 101 participants in the vaccine group on day 2, 19 (19%) of 101 participants on day 3, and one (1%) of 101 participants on day 5 (table 3). Viral isolation in culture was positive in six (6%) of 101 participants in the vaccine group on day 2. No virus was detected for day 3 and day 5 in the vaccine group. After the second vaccination, 40 (40%) of 101 participants on day 2 and nine (10%) of 101 participants on day 3 were positive by PCR; none of 101 participants were positive by PCR on day 5, and viral isolation was positive in only two out of 98 participants on day 2 in the vaccine group (2%; table 3). No virus was detected in all 98 participants on days 3 and 5. The median duration of viral shedding was 1 day. No viral RNA was detected in the placebo group after either the first or the second vaccination, indicating that LAIV H5N2 virus was not transmitted in the isolation ward. Sequence analysis of eight segment genes of virus isolated from nasal swab revealed four mutations, which were located on *NP, NA*, and *PB1* genes (appendix). None of these mutations have been shown to be related to the phenotype of vaccine virus.

**Table 3:** Detection of virus from nasal swabs of participants who had received A/turkey/Turkey/05/133 H5N2 LAIV vaccine candidate (n=101)

	**Participants positive after first immunisation (%)**	**Participants positive after second immunisation (%)**
**Viral culture**		
Day 2	6 (6%)	2 (2%)
Day 3	0	0
Day 5	0	0
**RT-PCR**		
Day 2	56 (55%)	40 (40%)
Day 3	19 (19%)	9 (10%)
Day 5	1 (1%)	0

Vaccination took place on day 1.

The H5N1 inactivated vaccine was safe; the most frequent local reactions were pain at the injection site and limitation of arm movement. Five (13%) of 40 participants in the vaccinated group and one (5%) of 20 participants in the naive group noticed moderate pain at the injection site, and six (15%) of 40 participants in the vaccinated group experienced a limitation in arm movement in the vaccinated group. Mild headache was reported by four (10%) of 40 participants in the vaccinated group and fatigue was reported in 11 (28%) of 40 participants in the vaccinated group. In the naive group, four (20%) of 20 participants experienced mild headache and five (25%) of 20 participants had fatigue. Local and systemic reactogenicity did not differ substantially between participants who had previously received the LAIV H5N2 group and those who were LAIV H5N2 vaccine-naive (appendix).

No pre-existing antibodies against H5N2 virus were detected in any participants before the first vaccination. After receiving two doses of LAIV H5N2 vaccine, 13 (13%) of 100 participants in the vaccine group had a four-fold or greater increase in antibody titre against H5N2 at any day, as measured by haemagglutination-inhibition assay (GMT 3·67 [95% CI 3·24–4·15]); detailed GMTs after each immunisation are in the appendix. Four-fold increases in neutralising antibodies were also detected in four (4%) of 100 participants in the vaccine group after the second dose (GMT 5·78 [5·34–6·26]). None of the participants in the placebo group had haemagglutination-inhibition (p=0·0051) or neutralising antibodies (p=0·303) against H5N2 virus. Four-fold increases in serum IgA were seen in 14 (14%) of 101 participants in the vaccinated group and none of 51 participants in the control group. A four-times increase in serum IgG was reported in 12 (12%) of 101 in the vaccinated group and none of 51 in the control group (appendix).

The haemagglutination-inhibition and micro-neutralisation antibody responses among participants in part 2 are shown in table 4. 39 (98%) of 40 participants who had previously received H5N2 LAIV developed a four-fold or greater rise in haemagglutination inhibition and 38 (95%) of 40 participants had a four-fold or greater rise in microneutralisation antibodies at day 7. All participants had a four-fold or higher increase than those in the naive group on day 28; peak haemagglutination-inhibition GMT was 566·89 and microneutralisation antibodies GMT was 1395·85 (p<0·0001 for both assays *vs* placebo). Peak GMT by haemagglutination-inhibition assay remained high up to day 90; 245·11 (95% CI 183·44–327·53) for the vaccine group and 26·39 (95% CI 13·52–51·52) for the naive group (p<0·0001). Peak GMTs for microneutralisation antibodies were 526·35 (371·13–746·49) for the vaccine group and 28·28 (16·80–47·62) for the naive group (p<0·0001).

**Table 4:** Serum antibody responses by influenza strain after inactivated H5N1 vaccine boosting between participants who had previously received vaccination (LAIV H5N2 vaccine; n=40) and participants who were naive (placebo; n=20) in the ITT population

		**Before vaccination**		**7 days after vaccination**		**28 days after vaccination**		**90 days after vaccination**	
		Seroconversion	GMT	Seroconversion	GMT	Seroconversion	GMT	Seroconversion	GMT
**Haemagglutination-inhibition antibodies**									
A/turkey/Turkey/05/133									
	Vaccinated	0/40 (0%; 0·00–0·00)	3·92 (3·24–4·75)	39/40 (98%; 92·66–100·00)	211·12 (134·45–331·52)	40/40 (100%; 100·00–100·00)	566·89 (436·97–735·44)	39/40 (98%; 92·66–100·00)	245·11 (183·44–327·53)
	Naive	0/20 (0%; 0·00–0·00)	2·59 (2·41–2·78)	3/20 (15%; 0·00–30·65)	3·66 (2·60–5·15)	14/20 (70%; 49·92–90·08)	25·49 (11·82–54·96)	15/20 (75%; 56·02–93·98)	26·39 (13·52–51·52)
	p value	..	0·0019*	<0·0001†	<0·0001*	0·0008‡	<0·0001*	0·0031‡	<0·0001*
A/Thailand/1 (KAN-1)/04									
	Vaccinated	0/40 0%; 0·00–0·00)	2·59 (2·46–2·72)	35/40 (88%; 77·25–97·75)	32·49 (22·01–47·96)	40/40 (100%; 100·00–100·00)	98·49 (75·44–128·58)	38/40 (95%; 88·25–100·00)	30·64 (22·93–40·94)
	Naive	0/20 (0%; 0·00–0·00)	2·50 (··)	0/20 (0%; 0·00–0·00)	2·77 (2·46–3·12)	3/20 (15%; 0·00–30·65)	5·18 (2·90–9·25)	4/20 (20%; 2·47–37·53)	5·36 (3·48–8·26)
	p value	..	0·3254*	<0·0001†	<0·0001*	<0·0001†	<0·0001*	<0·0001†	<0·0001*
A/Indonesia/5/2005									
	Vaccinated	0/40 (0%; 0·00–0·00)	2·59 (2·46–2·72)	38/40 (95%; 88·25–100·00)	68·45 (42·91–109·17)	39/40 (98%; 92·66–100·00)	187·00 (133·79–261·38)	38/40 (95%; 88·25–100·00)	59·14 (43·08–81·19)
	Naive	0/20 (0%; 0·00–0·00)	2·68 (2·42–2·96)	2/20 (10%; 0·00–23·15)	4·51 (3·12–6·52)	14/20 (70%; 49·92–90·08)	9·01 (5·55–14·64)	12/20 (60%; 38·53–81·47)	8·71 (5·28–14·36)
	p value	..	0·4792*	<0·0001†	<0·0001*	0·0042‡	<0·0001*	0·0004‡	<0·0001*
A/Laos/Nong Khai/1/2007									
	Vaccinated	0/40 (0%; 0·00–0·00)	2·50 (··)	35/40 (87%; 77·25–97·75)	33·06 (22·34–48·93)	38/40 (95%; 88·25–100·00)	105·56 (77·53–143·73)	37/40 (93%; 84·34–100·00)	35·95 (27·17–47·58)
	Naive	0/20 (0%; 0·00–0·00)	2·50 (··)	0/20 (0%; 0·00–0·00)	2·59 (2·41–2·78)	3/20 (15%; 0·00–30·65)	4·35 (2·58–7·34)	4/20 (20%; 2·47–37·53)	4·51 (2·84–7·15)
	p value	..	1*	<0·0001†	<0·0001*	<0·0001†	<0·0001*	<0·0001†	<0·0001*
**Microneutralisation antibodies**									
A/turkey/Turkey/05/133									
	Vaccinated	0/40 (0%; 0·00–0·00)	8·56 (6·48–11·29)	38/40 (95%; 88·25–100·00)	528·93 (333·88–837·91)	40/40 (100%; 100·00–100·00)	1395·85 (1040·79–1872·03)	39/40 (98%; 92·66–100·00)	526·35 (371·13–746·49)
	Naive	0/20 (0%; 0·00–0·00)	2·68 (2·42–2·96)	3/20 (15%; 0·00–30·65)	3·42 (2·47–4·72)	15/20 (75%; 56·02–93·98)	17·41 (9·05–33·48)	17/20 (85%; 69·35–100·00)	28·28 (16·80–47·62)
	p value	..	<0·0001*	<0·0001†	<0·0001*	0·0028‡	<0·0001*	0·0351‡	<0·0001*
A/Thailand/1 (KAN-1)/04									
	Vaccinated	0/40 (0%; 0·00–0·00)	2·50 (··)	35/40 (88%; 77·25–97·75)	25·49 (17·90–36·30)	40/40 (100%; 100·00–100·00)	52·78 (38·92–71·57)	36/40 (90%; 80·70–99·30)	22·65 (17·17–29·88)
	Naive	0/20 (0%; 0·00–0·00)	2·50 (··)	0/20 (0%; 0·00–0·00)	2·50 (..)	3/20 (15%; 0·00–30·65)	3·19 (2·50–4·06)	0/20 (0%; 0·00–0·00)	3·19 (2·72–3·73)
	p value	..	1*	<0·0001†	<0·0001*	<0·0001†	<0·0001*	<0·0001†	<0·0001*
A/Indonesia/5/2005									
	Vaccinated	0/40 (0%; 0·00–0·00)	5·27 (4·45–6·24)	37/40 (93%; 84·34–100·00)	84·27 (53·58–132·54)	40/40 (100%; 100·00–100·00)	200·43 (145·11–276·83)	38/40 (95%; 88·25–100·00)	78·59 (55·30–111·69)
	Naive	0/20 (0%; 0·00–0·00)	3·92 (3·24–4·75)	1/20 (5%; 0·00–14·55)	5·00 (3·71–6·73)	7/20 (35%; 14·10–55·90)	10·00 (6·48–15·43)	6/20 (30%; 9·92–50·08)	10·00 (6·40–15·63)
	p value	..	0·0382*	<0·0001†	<0·0001*	<0·0001‡	<0·0001*	<0·0001†	<0·0001*
A/Laos/Nong Khai/1/2007									
	Vaccinated	0/40 (0%; 0·00–0·00)	4·43 (3·88–5·05)	35/40 (88%; 77·25–97·75)	47·57 (32·70–69·20)	40/40 (100%; 100·00–100·00)	113·14 (83·71–152·90)	35/40 (88%; 77·25–97·75)	48·64 34·97–67·64)
	Naive	0/20 (0%; 0·00–0·00)	4·51 (3·84–5·28)	1/20 (5%; 0·00–14·55)	6·16 (4·85–7·81)	3/20 (15%; 0·00–30·65)	9·66 (6·38–14·61)	4/20 (20%; 2·47–37·53)	8·71 (5·91–12·83)
	p value	..	0·8205*	<0·0001†	<0·0001*	<0·0001†	<0·0001*	<0·0001†	<0·0001*

Data are n/N (%; 95% CI) or mean (95% CI), unless otherwise specified. Two-sided p values less than 0·01 were deemed significant. If every participant had the same titre, no 95% CI was calculated. GMT=geometric mean titre. ITT=intention-to-treat.

* Wilcoxon rank sum test.

† χ^2^ test.

‡ Fisher's exact test.

All participants in the previously LAIV H5N2 vaccinated group developed a four-fold or greater increase in haemagglutination-inhibition and neutralising cross-reactive antibody titres against clade 1 H5N1 virus (A/Thailand/1 [KAN-1]/04) with peak GMTs of 98·49 (95% CI 75·44–128·58) for haemagglutination-inhibition and 52·78 (38·92–71·57) for neutralising antibody (both p<0·0001 *vs* naive participants; table 4). Haema-gglutination-inhibition and microneutralisation antibody reactivity against A/Indonesia/05/05 H5N1 clade 2.1.3.2 and A/Lao/Nong Khai/1/07 H5N1 clade 2.3.4 was also observed with very similar GMTs (table 4). 95–100% of H5N2 LAIV experienced participants developed a four-fold or greater increase of haemagglutination-inhibition and neutralising antibody against clade 2.1.3.2 and 2.3.4 H5N1 viruses.

**Figure 2:**
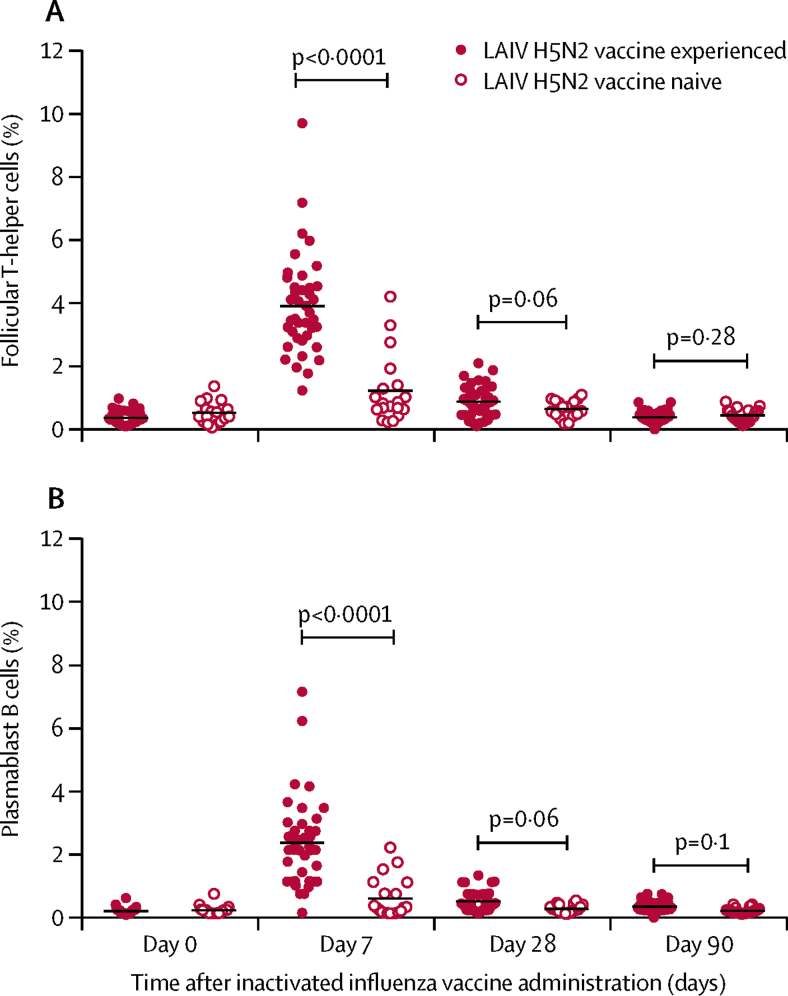
Follicular T-helper cells (A) and plasmablast cells (B) in circulating blood in participants who received inactivated H5N1 vaccine boost Follicular T-helper cells were defined as ICOS+CXCR3+CXCR5+CD4+ cells. Plasmablast cells were defined as CD19+CD3–CD20^lo^CD27+CD38+ cells.

The numbers of T_FH_ and plasmablast cells in blood increased on day 7 after the boosting vaccination (figure 2). The increase in plasmablast cells was strongly correlated with the increase in T_FH_ cells (*r*=0·813, p<0·0001). T_FH_ cell count was also correlated with the haemagglutination-inhibition titre (*r*=0·7044, p<0·0001) and microneutralisation titre (*r*=0·7165, p<0·0001), and there was a tendency for increased numbers of T_FH_ cells to be associated with increased antibody titres (appendix). However, no difference was recorded on days 28 and 90.

## Discussion

In this trial investigating the safety, immunogenicity, and priming effects of a live attenuated influenza candidate vaccine strain, we have shown that LAIV H5N2 vaccine was safe in healthy volunteers; both local and systemic reactogenicities were mild. The percentage of participants who reported local or systemic reactions after receiving LAIV H5N2 was lower than that reported in a study in Russia (10% *vs* 7% in our study). Virus shedding rate, as measured by viral culture 1 day after each immunisation, was also lower than that reported in the Russian trial (6%).^11^ However, this rate was similar to those reported in studies of using A/H5N1 LAIV strains based on A/Ann Arbor/6/60 H2N2.^9^ Low replication of viruses might be explained by the limited capacity of H5 viruses to bind to cells of the upper respiratory tract, because avian H5 viruses bind preferentially to sialic acid receptors terminating in N-acetylneuraminic acid α 2,3 galactose, whereas α 2,6 galactose receptors are predominant in human upper airway epithelium.^20^ Viral shedding after the second dose of LAIV H5N2 was strikingly lower than that seen after the first dose, suggesting that the first dose of LAIV H5N2 triggered an immune response, even though the concentrations of primary antibody were modest as measured by haemagglutination-inhibition assay. Thus, we anticipated that the chance of transmission is low. Apart from the above reason, all participants who were given placebo had negative nasal swabs for virus by both culture and PCR methods.

The inactivated H5N1 vaccine used in this study was found to be safe in both LAIV H5N2-vaccinated and vaccine-naive participants, similar to results from previous studies.^12^

Seroconversion, as detected by haemagglutination-inhibition and microneutralisation assays, after two doses of LAIV H5N2 vaccine in the Thai population in our study, was lower than that observed in the Russian population (14% *vs* 38%).^11^ This finding might be due to different assay procedures, because no global standard operating procedures exist, or the different ethnic groups studied. However, our study showed a similar proportion of seroconversions for the haemagglutination-inhibition assay compared with a small study.^21^

This study has shown that two intranasal doses of LAIV H5N2 followed by an H5N1 boost can induce a robust immune response to avian influenza H5N1 virus. In agreement with a previously reported smaller study,^16^ we detected high titres and rapid antibody responses after a single dose of inactivated H5N1 influenza vaccine given 1 year later in 100% of participants who had previously been vaccinated with LAIV H5N2. However, the antibody responses were similar to those observed in the earlier study, in which the inactivated vaccine was given 5 years later (roughly 87% seroconversion rate).^16^

These data suggest that LAIV vaccination induces long-lasting memory immune responses, and that these responses can be reactivated by inactivated H5N1 vaccine given 1–5 years later. The rapid antibody response in LAIV H5N2-experienced participants after receiving inactivated H5N1 vaccine (as early as 7 days) suggests that LAIV could be used to prime a population early during a pandemic, with the option to introduce the appropriate inactivated vaccine when it becomes available. It is also likely that the priming effect of LAIV H5N2 would permit an accelerated immune response in the case of infection with an H5 pandemic virus, providing a level of protection and control that could limit the spread of infection. However, several aspects need to be investigated further: the interval between administration of the LAIV prime and the inactivated vaccine boost, the combination of prime–boost strategies with different vaccines, the mechanisms underlying induction of an immune response by LAIV vaccine, and the quality of the cell-mediated immune response in the prime–boost setting.

Induction of cross-protective antibodies is very important for H5 vaccine development. The findings of this study showed that the antibodies detected in LAIV-experienced vaccinees after administration of inactivated H5N1 vaccine also cross-react with H5N1 virus circulating in Thailand (A/Thailand/1[KAN-1]/04; H5N1), which is genetically similar to clade 1 A/Vietnam/1203/04 H5N1 virus and H5N1 (clade 2.1.3.2 and 2.3.4 viruses).^22^ These data also indicate that giving the LAIV H5N2 vaccine would induce immune protection against different clades of H5 viruses, and that priming with H5N2 LAIV followed by boosting with H5N1 inactivated influenza vaccine elicits broad antibody responses, in terms of frequency and magnitude.

The main potential bias of part 2, which was a non-randomisation study by contrast with part 1 which was a randomised, controlled trial, could be the voluntary bias of the study participants. The characteristics of each group were similar, but there might be unknown confounders that could influence the study outcomes. Furthermore, the sample size of part 2 was calculated retrospectively as we were unable to predict how many participants could be contacted after the conclusion of part 1.

A subset of circulating ICOS+CXCR3+CXCR5+ T_FH_ cells was reported to play a crucial part in inducing antibody responses in seasonal influenza vaccine trials.^23^ This study showed a dramatic increase in T_FH_ cells in the LAIV-experienced group, but not in the LAIV-naive group, as early as 7 days after administration of inactivated H5 vaccine. This study clearly indicates that the H5N2 LAIV induced immune memory, and could partly explain the rapid antibody responses on boosting with inactivated vaccine. The increase in T_FH_ was strongly correlated with an increased number of plasmablast cells, suggesting that T_FH_ cells might have a key role in immunological events associated with induction of antibody responses. However, the numbers of T_FH_ and plasmablast cells were reduced on day 28 and day 90 after boosting vaccination. Our observation was consistent with a previous study,^23^ which showed a peak emergence of T_FH_ and plasmablast cells in blood on day 7 after boosting vaccination, which declined over the time. The cellular immune responses elicited by influenza specific interferon-γ producing T cells warrants further investigation.

Our study was done using a prime–boost strategy which, to our knowledge, has not been used in Thailand. The results provided from this study should be further evaluated for effectiveness if this strategy is applied in the field as a potential solution for pandemic control.
