# Supplementary material for: Safety and immunogenicity of a live attenuated influenza H5 candidate vaccine strain A/17/turkey/Turkey/05/133 H5N2 and its priming effects for potential pre-pandemic use: a randomised, double-blind, placebo-controlled trial
Source: Lancet Infect Dis. 2017 Aug;17(8):833–42. doi: 10.1016/S1473-3099(17)30240-2 (PMC5522535; doi:10.1016/S1473-3099(17)30240-2)
Supplement: Supplementary appendix [file mmc1.pdf]

THE LANCET
Infectious Diseases

Supplementary appendix

This appendix formed part of the original submission and has been peer reviewed. We post it as supplied by the authors.

Supplement to: Pitisuttithum P, Boonnak K, Chamnanchanunt S, et al. Safety and immunogenicity of a live attenuated influenza H5 candidate vaccine strain A/17/turkey/Turkey/05/133 H5N2 and its priming effects for potential pre-pandemic use: a randomised, double-blind, placebo-controlled trial. *Lancet Infect Dis* 2017; published online May 19. http://dx.doi.org/10.1016/S1473-3099(17)30240-2.

Supplementary Table 1: Local reaction following boosting vaccination with H5N1 inactivated vaccine in LAIV H5N2 experienced (N=40) and naïve (N=20) participants

Local Reaction*	Vaccinated group<br>N(%)	Naïve<br>N(%)
**Pain**		
Grade 0	14 (35.0)	9(45.0)
Grade 1	21(52.5)	10(50.0)
Grade 2	5(12.5)	1(5.0)
Grade 3	0(0.0)	0(0.0)
**p-value**	0.7295[1]	
**Swelling**		
Grade 0	36(90.0)	18(90.0)
Grade 1	3(32.5)	2(10.0)
Grade 2	1(2.5)	0(0.0)
Grade 3	0(0.0)	0(0.0)
**p-value**	1.000[1]	
**Limitation of arm movement**		
Grade 0	21(52.5)	15(75.0)
Grade 1	13(32.5)	5(25.0)
Grade 2	6(15.0)	0(0.0)
Grade 3	0(0.0)	0(0.0)
**p-value**	0.1113[1]	
**Erythema**		
Grade 0	40(100.0)	19(95.0)
Grade 1	0(0.0)	1(5.0)
Grade 2	0(0.0)	0(0.0)
Grade 3	0(0.0)	0(0.0)
**p-value**	0.3333[1]	
**Induration**		
Grade 0	40(100.0)	20(100.0)
Grade 1	0(0.0)	0(0.0)
Grade 2	0(0.0)	0(0.0)
Grade 3	0(0.0)	0(0.0)
**p-value**	-[2]	

* on any day

[1]Overall p-value (2-side) base on Fisher's exact test [2]no statistic was considered

Grade 0, no symptoms, Grade 1; mild symptoms, no significant interference with daily activity, Grade 2; moderate symptoms, affecting normal daily activity, Grade 3; severe symptoms markedly affecting normal daily activity

Supplementary Table 2: Systemic reaction following boosting vaccination with H5N1 inactivated vaccine in LAIV H5N2 experienced (N=40) and naïve (N=20) participants

Systemic Reaction*	Vaccinated group N(%)	Naïve N(%)
**Headache**		
Grade 0	34 (85.0)	16(80.0)
Grade 1	4(10.0)	4(20.0)
Grade 2	1(2.5)	0(0.0)
Grade 3	1(2.5)	0(0.0)
**p-value**	0.7479[1]	
**Fatigue**		
Grade 0	28(70.0)	15(75.0)
Grade 1	11(27.5)	5(25.0)
Grade 2	1(2.5)	0(0.0)
Grade 3	0(0.0)	0(0.0)
**p-value**	1.0000[1]	
**Chills**		
Grade 0	37(92.5)	20(100.0)
Grade 1	3(7.5)	0(0.0)
Grade 2	0(0.0)	0(0.0)
Grade 3	0(0.0)	0(0.0)
**p-value**	0.5441[1]	
**Dizziness**		
Grade 0	36(90.0)	18(90.0)
Grade 1	3(7.5)	2(10.0)
Grade 2	1(2.5)	0(0.0)
Grade 3	0(0.0)	0(0.0)
**p-value**	1.0000[1]	
**Runny nose**		
Grade 0	37(97.5)	20(100.0)
Grade 1	0(0.0)	0(0.0)
Grade 2	1(2.5)	0(0.0)
Grade 3	0(0.0)	0(0.0)
**p-value**	1.0000[1]	

Systemic Reaction*	Vaccinated group N(%)	Naïve N(%)
**Sore throat**		
Grade 0	38(95.0)	20(100.0)
Grade 1	2(5.0)	0(0.0)
Grade 2	0(0.0)	0(0.0)
Grade 3	0(0.0)	0(0.0)
**p-value**	0.5480[1]	
**Cough**		
Grade 0	35(87.5)	20(100.0)
Grade 1	5(12.5)	0(0.0)
Grade 2	0(0.0)	0(0.0)
Grade 3	0(0.0)	0(0.0)
**p-value**	0.1588[1]	
**Myalgia**		
Grade 0	38(95.0)	20(100.0)
Grade 1	2(5.0)	0(0.0)
Grade 2	0(0.0)	0(0.0)
Grade 3	0(0.0)	0(0.0)
**p-value**	0.5480[1]	
**Arthralgia**		
Grade 0	39(97.5)	20(100.0)
Grade 1	1(2.5)	0(0.0)
Grade 2	0(0.0)	0(0.0)
Grade 3	0(0.0)	0(0.0)
**p-value**	1.0000[1]	
**Nausea**		
Grade 0	38(95.0)	20(100.0)
Grade 1	2(5.0)	0(0.0)
Grade 2	0(0.0)	0(0.0)
Grade 3	0(0.0)	0(0.0)
**p-value**	0.5480[1]	
**Vomiting**		
Grade 0	40(100.0)	20(100.0)
Grade 1	0(0.0)	0(0.0)

Systemic Reaction*	Vaccinated group N(%)	Naïve N(%)
Grade 2	0(0.0)	0(0.0)
Grade 3	0(0.0)	0(0.0)
**p-value**	-[2]	
**Poor appetite**		
Grade 0	40(100.0)	20(100.0)
Grade 1	0(0.0)	0(0.0)
Grade 2	0(0.0)	0(0.0)
Grade 3	0(0.0)	0(0.0)
**p-value**	-[2]	
**Diarrhea**		
Grade 0	40(100.0)	20(100.0)
Grade 1	0(0.0)	0(0.0)
Grade 2	0(0.0)	0(0.0)
Grade 3	0(0.0)	0(0.0)
**p-value**	-[2]	
**Rash**		
Grade 0	40(100.0)	20(100.0)
Grade 1	0(0.0)	0(0.0)
Grade 2	0(0.0)	0(0.0)
Grade 3	0(0.0)	0(0.0)
**p-value**	-[2]	

* on any day

[1]Overall p-value (2-side) base on Fisher's exact test [2]no statistic was considered

Grade 0, no symptoms, Grade 1; mild symptoms, no significant interference with daily activity, Grade 2; moderate symptoms, affecting normal daily activity, Grade 3; severe symptoms markedly affecting normal daily activity

Supplement Table 3: Sequence analysis of eight segment genes of virus isolated from nasal swabs

Participant ID	Visit	Passage in eggs	HAI titer	Nucleotide/Amino acid substitution							
				NP	NA	PB1	PB2	PA	HA	NS	M
9-1-1-007	Day 2	1	128	**G**AT100/**A**AT (D34N)	-	-	-	-	-	-	-
9-1-1-012	Day 2	1	1024	-	GC**A**399/GC**G** (A133A)	-	-	-	-	-	-
9-1-1-019	Day 2	1	256	-	-	-	-	-	-	-	-
9-1-1-022	Day 2	1	512	-	-	AA**G**615/AA**T** (K205N)	-	-	-	-	-
9-1-1-024	Day 2	1	1024	-	-	-	-	-	-	-	-
9-1-1-024	Day 22	1	64	-	-	A**A**G665/A**G**G (K222R)	-	-	-	-	-

Supplement Table 4: Serum antibody responses against A/17/turkey/Turkey/05/133 (H5N2) on days 1,7,28 and 90 with peak GMT following two doses LAIV H5N2 vaccine or placebo (modified ITT population)

Assay	Study group(n)	Prior to vaccination		28 days after vaccination		49 days after vaccination		60 days after vaccination	
		Percentage of subjects with 4-fold Ab rise n/N % (95% CI)	GMT (95% CI)	Percentage of subjects with 4-fold Ab rise n/N % (95% CI)	GMT (95% CI)	Percentage of subjects with 4-fold Ab rise n/N % (95% CI)	GMT (95% CI)	Percentage of subjects with 4-fold Ab rise n/N % (95% CI)	GMT (95% CI)
HAI	Vaccinated (N= 101, n = 100)	0 (0.00)	2.50 (-)	3 (3.00)	2.68 (2.52 - 2.85)	13 (13.00)	3.64 (3.22 - 4.12)	13 (13.00)	3.67 (3.24 - 4.15)
	Placebo (N= 51, n = 49)	0 (0.00)	2.50 (-)	0 (0.00)	2.50 (-)	0 (0.00)	2.50 (-)	0(0.00)	2.50 (-)
	p-value	- [3]	1.0000[5]	0.5511[1]	0.0822[5]	0.0051[1] *	<0.0001[5] *	0.0051[1] *	<0.0001[5] *
MN	Vaccinated (N= 101, n = 100)	0 (0.00)	5.00 (-)	2 (2.00)	5.18 (4.93 - 5.44)	4 (4.00)	5.82 (5.38 - 6.31)	4 (4.00)	5.78 (5.34 - 6.26)
	Placebo (N= 51, n = 49)	0 (0.00)	5.00 (-)	0 (0.00)	5.00 (-)	0 (0.00)	5.00 (-)	0 (0.00)	5.00 (-)
	p-value	- [3]	1.0000[5]	1.0000[1]	0.3255[5]	0.3030[1]	0.0032[5] *	0.3030[1]	0.0045[5] *

Note: SID 921075, 921084, 921089 are missing value at Day 1, 28, 49 and 60 on serum IgA and IgG.
SID 921075, 921084, 921089 are missing value at Day 28, 49 and 60 on HAI and Micro NT.
[1] Overall p-value (2-sided) based on Fisher's exact test.
[2] Overall p-value (2-sided) based on Chi-square test.
[3] No statistic was considered.
[4] Overall p-value (2-sided) based on Independence T-Test
[5] Overall p-value (2-sided) based on Wilcoxon rank sum test
* significant difference

Supplement Table 5. Antibody responses following two doses of LAIV H5N2 vaccine or placebo

Antibody assay	Study group	Fourfold raised titre on any day No. (%)	Twofold raised titre on any day No. (%)	Peak GMT (95% CI)
**Serum IgA**	Vaccine (n = 101)	14 (13.9)	54 (53.5)	326.7 (268.8–397.1), n = 67
	Placebo (n = 51)	0 (0.0)	18 (35.3)	226.3 (152.1–336.7), n = 28
	p value =	0.0050[1] *	0.0559[1]	0.0482[4] *
**Serum IgG**	Vaccine (n = 101)	12 (11.9)	58 (57.4)	1061.65 (846.4–1331.6), n = 63
	Placebo (n = 51)	0 (0.0)	5 (9.8)	621.78 (464.1–833.0), n = 24
	p value =	0.0090[1] *	<0.0001[1] *	0.0042[4] *

[1] Overall p value (2-sided), based on Fisher's exact test.
[2] Overall p value (2-sided), based on Chi-squared test.
[3] No statistic was considered.
[4] Overall p value (2-sided), based on Wilcoxon rank sum test.
* Significant difference, Analysis as ITT

Supplementary Figure 1. Correlation of TFH cells after inactivated H5N1 vaccination in LAIV H5N2-experienced group with: A, number of plasmablast cells; B, HAI antibody titres; C, neutralizing antibody titres.

A.

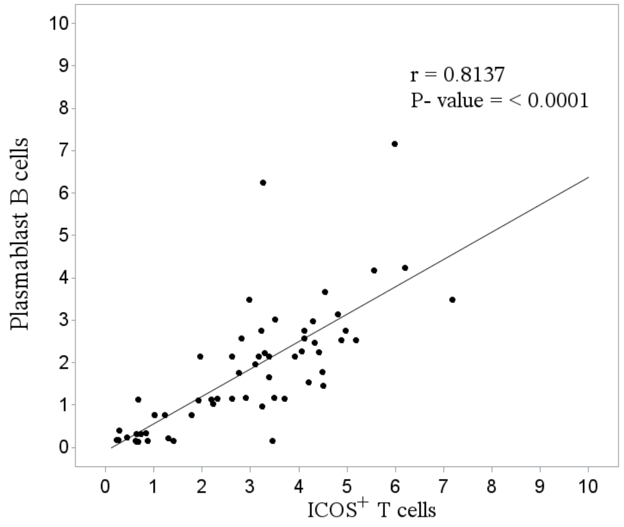

B.

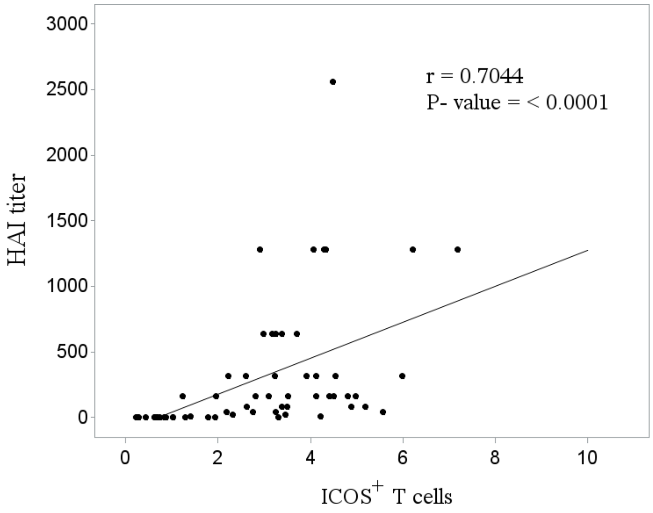

C.

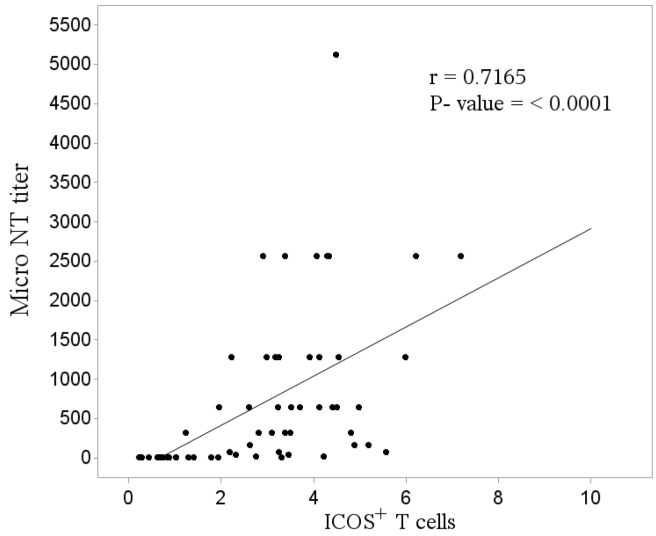

r = 0.7165
P- value = < 0.0001
Micro NT titer
ICOS+ T cells
0
500
1000
1500
2000
2500
3000
3500
4000
4500
5000
5500
0
2
4
6
8
10
